# QCM-based immunosensor for rapid detection of *Salmonella* Typhimurium in food

**DOI:** 10.1038/s41598-018-34285-y

**Published:** 2018-10-31

**Authors:** Andrea Fulgione, Martina Cimafonte, Bartolomeo Della Ventura, Marco Iannaccone, Concetta Ambrosino, Federico Capuano, Yolande Thérèse Rose Proroga, Raffaele Velotta, Rosanna Capparelli

**Affiliations:** 1 Department of Agriculture, University of Naples “Federico II”, Portici (Naples), 80055 Italy; 2 Department of Science and Technology, University of Sannio, Benevento, 82100 Italy; 3 Department of Physics “Ettore Pancini”, University of Naples “Federico II”, Naples, 80126 Italy; 4 Department of Food Inspection, Istituto Zooprofilattico Sperimentale del Mezzogiorno, Portici (Naples), 80055 Italy

## Abstract

*Salmonella* Typhimurium is one of the main causes of outbreaks and sporadic cases of human gastroenteritis. At present, the rapid detection of this pathogen is a major goal of biosensing technology applied to food safety. In fact, ISO standardized culture method takes up to ten days to provide a reliable response. In this paper, we describe a relatively simple protocol for detecting *Salmonella* Typhimurium in chicken meat based on a Quartz-Crystal Microbalance (QCM), which leads to a limit of detection (LOD) less than of 10° CFU/mL and requires a pre-enrichment step lasting only 2 h at 37 °C. The reliability of the proposed immunosensor has been demonstrated through the validation of the experimental results with ISO standardized culture method. The cost-effectiveness of the procedure and the rapidity of the QCM-based biosensor in providing the qualitative response make the analytical method described here suitable for applications in food inspection laboratory and throughout the chain production of food industry.

## Introduction

*Salmonella* species are facultative anaerobic Gram-negative bacteria^1^. Worldwide, they are foodborne pathogens and second only to *Campylobacter spp*. for causing gastrointestinal human infections^2^. In the EU member states 6.2 million cases of human salmonellosis occur each year^3^. In particular, *Salmonella* Typhimurium (*S*. Typhimurium) represents one of the main pathogens responsible for provoked human gastroenteritis^4^, which is caused mainly by the consumption of raw or uncooked eggs, poultry meat vegetables, fruits and foodstuff of animal origin^5–8^. Infection symptoms such as fever, nausea, vomiting, diarrhea, dehydration, weakness, and loss of appetite usually appear 12–72 h after ingestion of contaminated products. Several studies focused on the control of *Salmonella* contaminations by the use of new natural antimicrobial molecules for controlling bacteria contaminations^9,10^, but the crucial step represented by the rapid detection of pathogen in the food still remains.

The official food safety agencies - such as US Food and Drug Administration (FDA) and International Organization of Standardization (ISO)^11^ – recommend the conventional culture method as the reference test for food analyses. The ISO method includes pre-enrichment steps followed by selective enrichment, isolation on selective agars, biochemical characterization of probable colonies, and their final serological confirmation. Inevitably, the positive time response with this procedure, ranges from five to about ten days. This method is very sensitive and cheap; however, it is labor-intensive, time-consuming and unsuitable for testing a large number of samples. While waiting for test results without shipping the foodstuffs could have a strong negative impact on the business profitably, the alternative of missing possible positive result for pathogenic contamination, obviously, implicates costly recalls of goods, human suffering and loss of reputation^12^.

In this context, the development of rapid, cost-effective, and automated methods for the identification and, eventually, quantification of bacteria such as *S*. Typhimurium is urgent. These methods, - integrated with preventive strategies such as Hazard Analysis Critical Control Points (HACCP) - could significantly improve the safety of the food chain, from raw to processed foods^13^. Such an issue has been addressed by adopting a number of strategies^14^, but the goal is far from being accomplished since the current regulation for food analysis requires that alternative methods, should have sensitivity and specificity of at least 99%, be rapid and not requiring specialized personnel to carry out the analysis^15^. These techniques can be grouped as immunology-based assays (ELISA), nucleic acid-based (PCR) assays, and biosensors. The first two groups have specificity and sensitivity almost comparable to the conventional methods^14,16^, but cannot be considered easy-to-use techniques. Moreover, they share with other tests for *Salmonella spp*. some drawbacks such as the need for a long pre-enrichment step to recover stressed cells, possible cross-reactions with related antigens, sensitivity and antigen modification depending on the sample matrix, and high cost for automation and industrial application. Furthermore, the result provided by these methods is easily influenced by intrinsic characteristics of the food such as background microbiota, sample matrix and inhibitory substances (heavy metals, proteins, fats, carbohydrates, antibiotics and organic compounds)^17^. In addition, though PCR is very sensitive, thereby allowing shorter enrichment times, it is prone to detect also dead bacteria.

In contrast to complex and lengthy methods, biosensors offer accurate and rapid detection method for pathogens in foodstuffs so to be applicable on a wider scale as, for instance, in selecting the correct direction for finished products: sale at retail market or industrial treatments to eliminate *Salmonella* bacteria^15^. Currently, considerable attention is given to rapid and sensitive biosensor devices based on surface plasmon resonance (SPR)^18–20^ and photoelectrochemical immunoassay (PEC)^21–24^. Together with enzyme controlled colorimetric sensor^25^, all these devices are able to detect very low concentrations of different molecules and proteins with an acceptable specificity and reproducibility, but it is worth to highlight that these classes of sensors still requires specialized personnel in view of the complexity of the surface functionalization procedure^26^. Electrochemical-based biosensors exploit the biological recognition of compounds like enzymes, antibodies, DNA and aptamers. This class of biosensors can reach wide linear ranges (10-10^5^ CFU/mL) and good LOD (5 CFU/mL), although their application to food samples is limited by cross-reactivity^27,28^. Rapid detection is also provided by QCM-based immunosensors that can be considered user-friendly, but they currently lack the required sensitivity unless ballasting procedures by antibodies or even by functionalized nanoparticles are carried out^29–33^. The application of such a procedure to bacteria has led to LODs of 10^2^ CFU/mL using ballasting procedure or QCM-based aptasensor and 10^1^ CFU/mL with gold nanoparticles^34,35^.

Here, we describe a simple, reliable, fast, sensitive and specific QCM-based immunosensor, which is able to detect concentration less than 10° CFU/mL of *S*. Typhimurium in chicken meat after 2 hours of pre-enrichment step at 37 °C, in compliance with ISO standardized culture method. The method we propose exploits an effective surface functionalization (PIT, photochemical immobilization technique) that is able to tether upright antibodies on gold surfaces, thereby enhancing the sensitivity of the device. We also show that the presence of food constituents does not prevent bacteria from being recognized by antibodies making our approach extendable to further applications.

## Results

### QCM detection of *S*. Typhimurium

**Figure 1:**
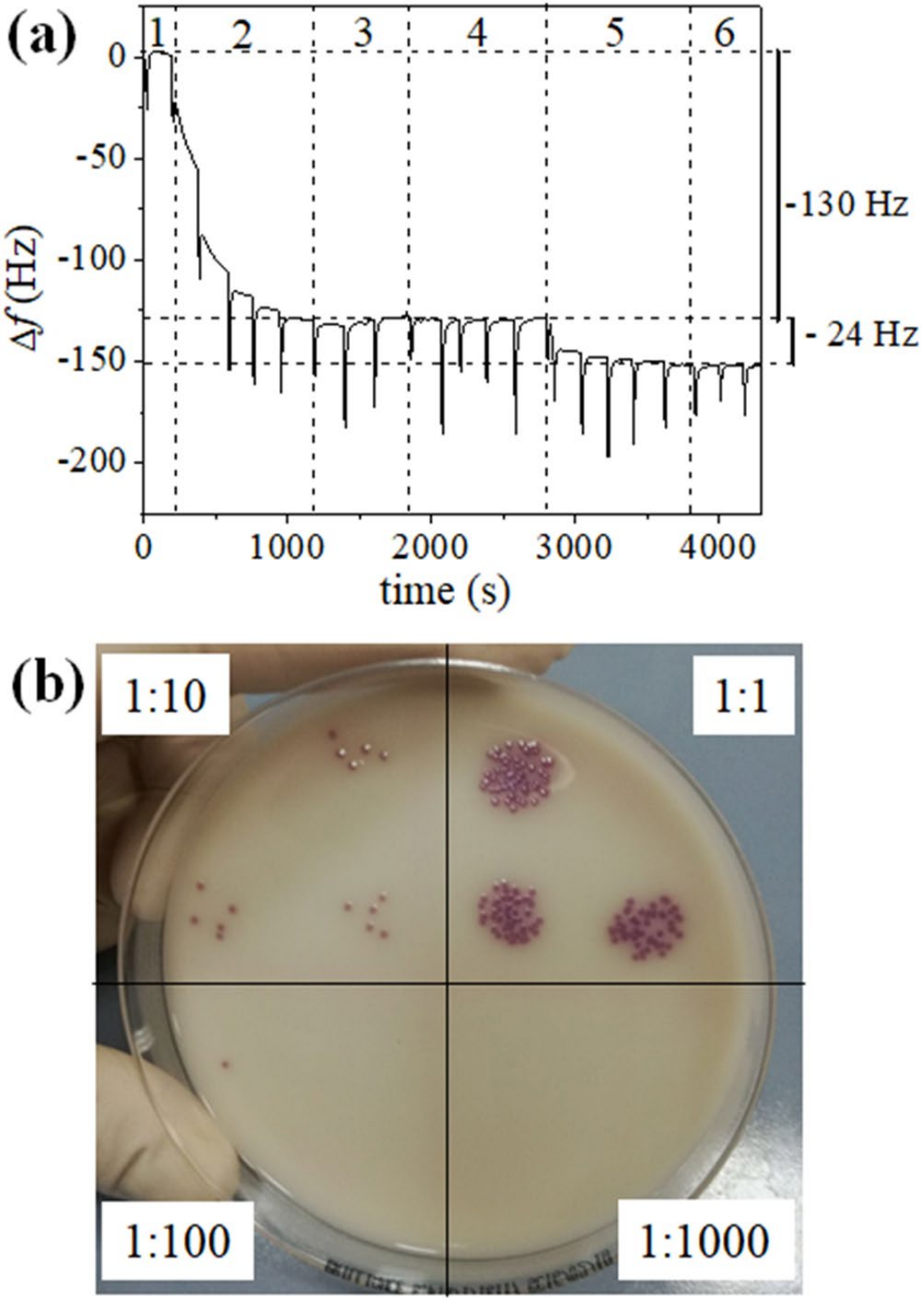
QCM sensorgram and microbiological detection of *S*. Typhimurium. Food sample contaminated with *Salmonella* Typhimurium (10^0^ CFU/mL). (**a**) QCM sensorgram after pre-enrichment step (2 h at 37 °C); (**b**) Spot dilutions of the sample before the pre-enrichment step on Salmonella Chromogenic Agar Base after overnight incubation. Each section shows the dilution factor.

QCM provides a frequency change proportional to the mass deposited on the surface^36,37^ and, a typical sensorgram is shown in Fig. 1a. The spikes in the plot are due to the syringe draws and do not affect the measurement of the frequency shift. The sensorgram refers to a chicken sample – initially contaminated with 10° CFU/mL of *S*. Typhimurium - after 2 h of pre-enrichment step at 37 °C, a time leading to approximately 10^2^ multiplication factor by assuming a replication time of 20 min^38^.The initial concentration was checked in three independent experiments by spotting 10 µL of the samples analysed by QCM at different dilutions. One of these cases is shown in Fig. 1b where the top left section (dilution 1:10) contains three spots each containing 5 CFU/10 μL. After the stabilization of the resonance frequency by PBS 1X solution (step 1 in Fig. 1a), the surface was functionalized (step 2) by conveying to the QCM, a solution containing UV activated anti-*Salmonella* polyclonal antibodies (anti-*Salmonella* pAbs; 50 µg/mL), which brings about a frequency drop of about 130 Hz. The subsequent washing step with PBS 1X (step 3) was carried out to remove possible anti-*Salmonella* pAbs not tethered onto gold surface, whereas blocking with BSA (step 4) was carried out to prevent non-specific signal from the uncovered gold surface. The lack of significant frequency change during the last step demonstrates that the whole electrode was covered with anti-*Salmonella* p-Abs. Eventually, step 5 and 6 correspond to the sample injection after 2 hours of pre-enrichment (10° CFU/mL initial concentration) and the final washing, respectively.

**Figure 2:**
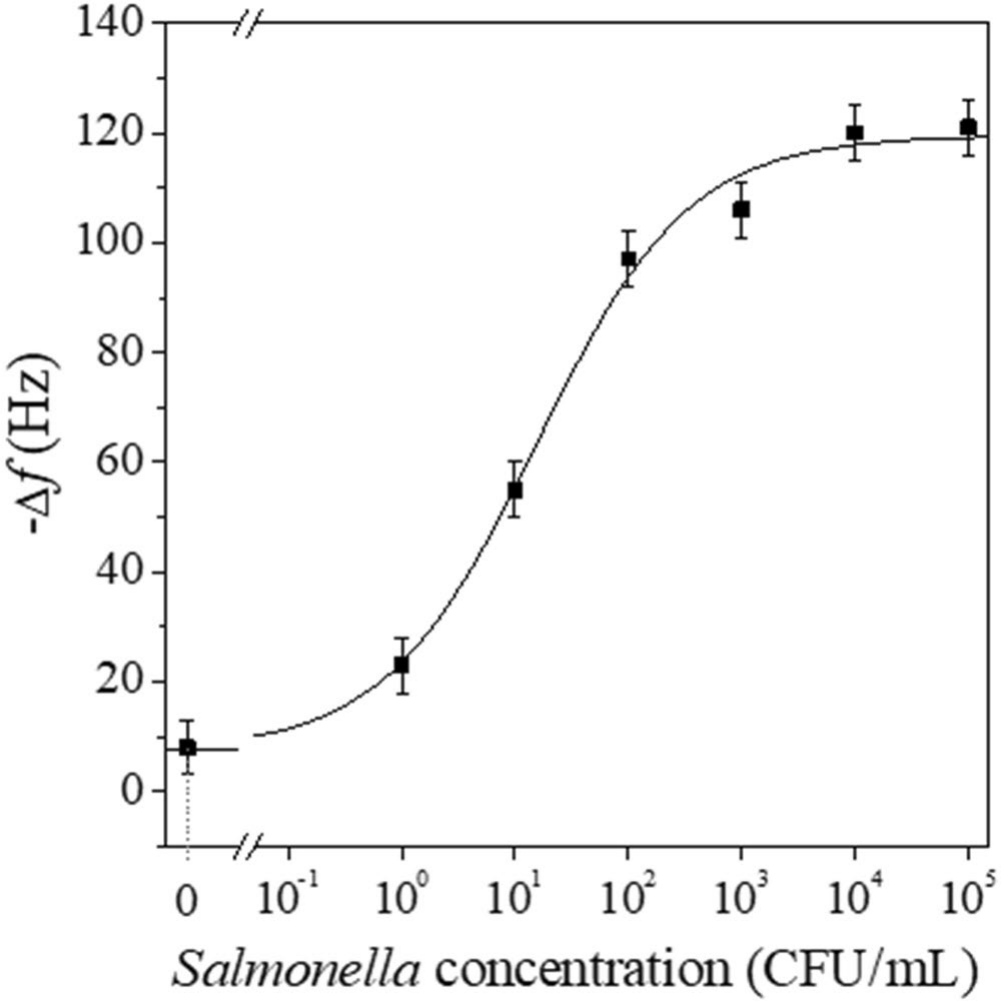
Dose-response curves of the QCM-based biosensor referred to samples of chicken meat contaminated with different concentrations of *Salmonella* Typhimurium. The curve is the best fits of the experimental values obtained by a logistic function.

In order to evaluate the performance of the QCM sensor, we measured the dose-response curve that is reported in Fig. 2, in which the values on *x*-axis refer to the initial *Salmonella* concentration selected for the contamination of chicken sample. The samples were analysed after 1 and 2 hours of pre-enrichment, but with 1 h pre-enrichment step, the immunosensor was able to detect *Salmonell*a only in the range 10^2^–10^5^ CFU/mL (data not shown), whereas 2 hours pre-enrichment allowed us to decrease the lower limit down to 10° CFU/mL (see Fig. 2).

With the longer pre-enrichment it was possible to observe a frequency shift induced by the step (5) of the protocol for all the samples infected, whereas no significant frequency shift could be detected with uncontaminated chicken sample (negative control). The subsequent washing (step 6) did not lead to any frequency change; thus, the lack of detachment from the surface underscores once again the stability of the interaction between bacteria and UV photoactivated anti-*Salmonella* pAbs and, hence, demonstrates the effectiveness of PIT as a gold surface functionalization procedure. Each point of the dose-response curve (Fig. 2) represents the mean triplicates of measurements carried out in several days, in different environmental conditions, and using different electrodes.The curve displays a linearity range of three decades from 10° to 10^3^ CFU/mL before reaching a saturation profile at the highest concentrations, whereas the frequency shift measured for the negative sample was 7.5 ± 4.0 Hz and is may well be due to non-specific interaction of the food constituents with the biosensor surface. Such a value for the blank does not prevent us from estimating a very low limit of detection of our biosensing procedure. In fact, the concentration that provides a signal corresponding to 3 SD of the background noise (7.5 Hz + 3 × 4.0 Hz ≈ 20 Hz) is lower than 10° CFU/mL (Fig. 2), a concentration that can be safely considered the LOD of the proposed biosensor.

### Specificity test

**Figure 3:**
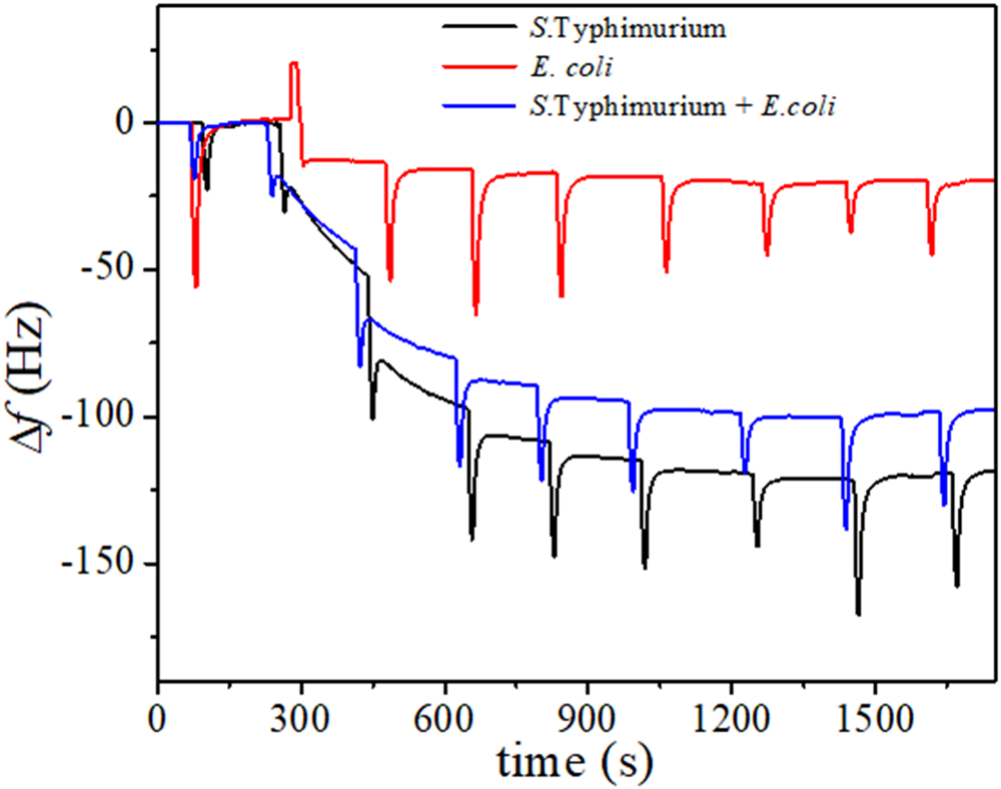
QCM biosensor specificity. Sensorgrams of *Salmonella* Typhimurium (black curve), *Escherichia coli* (red curve) and both bacteria (blue curve) contaminated chicken meat samples, after the pre-enrichment step (2 h at 37 °C). Each bacterial concentration was at 10^4^ CFU/mL before the pre-enrichment step.

The specificity tests have been carried by considering the possible cross-interference provided by *Escherichia coli* (*E. coli*), which is among the most spread bacteria in food. Figure 3 shows the sensorgrams measured when chicken meat samples were contaminated by *S*. Typhimurium, *E. coli* and a mixture of them. In all the cases, the initial concentration of each bacteria was 10^4^ CFU/mL and the pre-enrichment step lasted 2 hours at 37 °C. It was observed a significant frequency variation only when *Salmonella* bacteria were present (Fig. 3; black or blue lines), whereas no significant variation was observed when the sample was contaminated with only *E. coli* (Fig. 3; red line). In particular, the frequency detected in the case of *Salmonella* chicken meat sample (115 Hz; black line) was comparable to that reported on dose response curve. Instead, the drop frequency evaluated in samples infected with both *S*. Typhimurium and *E. coli* was lower respect to that of only *Salmonella* infected sample (95 Hz (blue line) vs 115 Hz (black line)). This last result can be ascribed to the competition - between the two bacteria during the enrichment step, when both are present in food matrix. The initial bacterial concentration selected for the test were also supported by microbial count carried out on the same sample and highlight once again the high specificity of the anti-*Salmonella* pAbs produced against *Salmonella* respect to *E. coli* even when they are photoactivated, demonstrating, by this way, the effectiveness of the proposed QCM sensor in detecting *Salmonella* in a real food matrix as chicken meat.

## Discussion and Conclusion

In summary, we put forward an easy-to-use method for detecting *S*. Typhimurium in a real food matrix (chicken meat) based on a QCM functionalized with anti-*Salmonella* polyclonal antibodies UV activated by PIT. This last technique represents a very important and innovative approach to improve biosensor sensitivity. Usually, Abs bind to a surface preferentially with Fab, assuming a bended conformation and exposing Fc region. This happened also in the case of no treated Abs on gold surface, thus limiting the interaction of the same Abs with the antigens. On the contrary, a recent study evidenced that, when Abs are irradiated prior their interaction with a surface - like gold -, they assumed a preferential orientation with one Fab domain protruding and the other two portions of the Ab interacting with surface^39^.

The QCM measurement procedure does not require specialised personnel, and it is rapid, low-cost, and suitable for out-of-lab use. The single analysis requires minimal sample treatment: pre-enrichment (2 h) at 37 °C, preparation steps (two centrifugations) and injection into the cell by five syringe draws, so that the whole measurement can be carried out in less than 4 hours. Although not suitable for accurate quantitative analysis, the QCM sensor proposed here lends itself as a very attractive device for “on-off” qualitative analysis of food contaminated. The LOD of proposed biosensor (less than 10^0^ CFU/mL of *S*. Typhimurium in chicken sample) and the minimum sample pre-enrichment step (2 h at 37 °C) required, make the biosensor suitable for food inspection when extremely low contamination levels need to be detected in chicken samples and, “rapidly” in test response “for all foodstuffs” respect to other diagnostic biosensors which, firstly, require a pre-enrichment step ranging from 6 up to 58 hours^15^.

The specificity of the whole system is more than satisfactory since no significant signal is detected when the *Salmonella* is replaced by *E. coli* while a signal is distinguished when both bacteria are present. The application of this biosensor to detect other foodborne pathogens represents a new alternative method to monitor the food safety throughout the chain production so warranting the final quality of the products. Furthermore, this biosensor satisfies some of the technical parameters such as detection limit, time of analysis, validation of the method, sensitivity and specificity, and additional parameters like equipment, operation, and costs which should be considered before adapting a new rapid detection method^15,40,41^.

## Methods

### Bacteria

*Salmonella enterica* subsp. enterica serovar Typhimurium ATCC 13311 (*S*. Typhimurium) and *E. coli* ATCC 25922 were used to carry out the chicken meat samples infection.

### Antibodies production and purification

Antibodies were produced in Fischer 344 rats (Harlan, Italy). Each animal received two intraperitoneal injections-at two weeks interval-of heat killed *S*. Typhimurium bacteria at 10^7^ CFU/mL in 0,9% NaCl (300 µL), emulsified with an equal volume of incomplete Freund’s adjuvant (Sigma-Aldrich, Italy). After 1 week from the last injection, animals were sacrificed, and peripheral blood was collected. Serum was separated by centrifugation at 5000 rpm for 5 min. IgG purification was carried out by using NAb™ Protein G Spin Kit (Thermo Fisher Scientific, USA). Anti-*Salmonella* pAbs level was determined by NanoDrop 2000 spectrophotometer (Thermo Fisher Scientific, USA). All procedures were approved by Italian Minister of Health (authorization n° 891/2015-PR) and were performed in accordance with relevant guidelines and regulations.

### Salmonella antibodies titer and immunoadsorption

In order to evaluate the cross-reactivity of anti-*Salmonella* pAbs against major foodborne pathogens, the antibodies titer was measured by ELISA. Briefly, 100 µl of *S*. Typhimurium (10^5^ CFU/mL) in pure PBS were seeded in a 96-well plate. After incubation overnight at room temperature, wells were washed 3 times with 0.05% Tween 20 (Sigma-Aldrich, Italy) (v/v) in pure PBS; quenched with 100 μL of 1% Bovine Serum Albumin (BSA; Sigma-Aldrich, Italy) (v/v) in pure PBS and incubated at room temperature for 1 h. Then, 100 μL of anti-*Salmonella* pAbs at different dilutions (1:20; 1:50; 1:60; 1:80; 1:100; 1:150) were added to the wells and the plate was incubated at room temperature for 3 h using a constant shaking (100 rpm). Subsequently the wells were rinsed 3 times with 0.05% Tween 20 (v/v) in pure PBS and incubated 1 h at room temperature with 100 µl of Goat Anti-Rat HRP (Abcam, UK) diluted 1:5000. Then, wells were washed 3 times with 0.05% Tween 20 (v/v) in pure PBS and was added 3,3′,5,5′-Tetramethylbenzidine (TMB; 50 μL; Sigma-Aldrich, Italy). The plate was incubated again, in the dark, at room temperature for 20 min. The reaction was stopped by the addition of 0.16 M H_2_SO_4_ (100 µl) to all wells and the titer was determined through the measurement of the optical absorbance at 450 nm (Biorad microplate reader model 680, USA).

**Figure 4:**
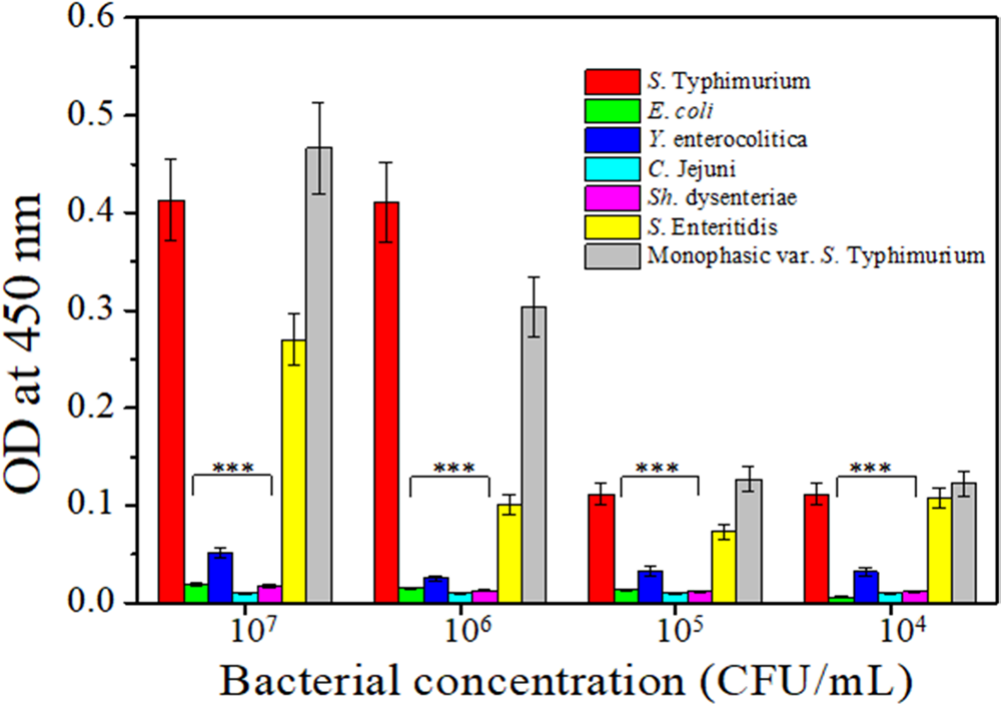
Anti–*Salmonella* pAbs specificity against different foodborne pathogens. Cross-reactivity between anti-*Salmonella* pAbs and selected foodborne pathogens: *Escherichia coli*, *Yersinia enterocolitica*, *Campylobacter jejuni*, *Shigella dysenteriae*, *Salmonella Enteritidis*, *Salmonella* Typhimurium and the monophasic variant of *Salmonella* Typhimurium. Results are presented as mean value ± S.D and are representative of three independent experiments, each performed in triplicate. ***p value < 0.001.

Subsequently, an immunoadsorption protocol was carried out to avoid any possible cross-reaction of anti-*Salmonella* pAbs with *E. coli* bacteria. Briefly, in 1.5 mL eppendorf tube, quenched with 2% skimmed milk powder (Sigma-Aldrich, Italy), 50 µl of pure PBS containing *E. coli* at the concentration of 10^8^ CFU/mL were mixed with 1 mL of anti-*Salmonella* pAbs diluted 1:50. The eppendorf was incubated at room temperature overnight, under constant shaking (100 rpm) and later centrifuged at 13400 rpm for 5 min. The supernatant, containing the antibodies which have not interacted with *E. coli*, was collected and the anti-*Salmonella* pAbs level was determined by NanoDrop 2000 spectrophotometer (Thermo Fisher Scientific, USA)^42–44^. The same titer above mentioned was also adopted to evaluate the cross-reactivity of anti-*Salmonella* pAbs with other foodborne pathogens such as *Yersinia enterocolitica, Campylobacter jejuni, Shigella dysenteriae, Escherichia* coli*, Salmonella* enteritidis, and the monophasic variant of *S*. Typhimurium (serotype 1,4,[5],12:i:-). The results (Fig. 4) evidence the high reactivity of anti-*Salmonella* pAbs against three different *Salmonella* strains compared to non-Salmonella strains (*p* < 0.001). Rather than a drawback, the significant reactivity with other *Salmonella* strains is a strength feature of our pAbs since both the monophasic variant of *S*. Typhimurium and *S*. Enteritidis are hazardous for human health, so that possible positive response of the biosensor coming from the presence of these two strains would be beneficial for food analysis in practical use.

### QCM immunosensor and measurement

The quartz oscillators (AT10-14-6-UP-01) were from Novaetech S.r.l. (Naples, Italy). They were AT-CUT quartz with a fundamental frequency of 10 MHz. The crystal and gold electrode diameters are 1.37 cm and 0.68 cm, respectively. The gold surfaces were cleaned by immersing the oscillators for 1 min in a glass beaker containing Piranha solution (3:1 ratio between concentrated sulfuric acid and 40% hydrogen peroxide solution); then, the quartzes were washed with milli-Q water. The whole cleaning procedure, strong enough to remove any organic residue from the gold surface, was performed in the hood and can be repeated 3-4 times before the quartz is exhausted and has to be changed. The piranha treatment requires new functionalization, a step which is cheap and quick when carried out by PIT. The gold-quartz wafer was placed on the electronic console and the resonance frequency of the oscillator was monitored by producer-released software. The QCM was integrated into a microfluidic circuit consisting of the cell which contains the oscillator, Tygon tubes, and a syringe. The volume of the circuit was about 200 μL and the syringe was used to suck a similar volume repeatedly. The syringe draws conveyed fresh solution to the cell and were separeted each other by a time interval of 3 min that showed to be long enough to reach a temporary frequency stabilization. No further frequency change was observed after 5 syringe draws so that 1 mL was the volume to be changed for reaching the equilibrium (frequency stabilization); since it was changed by sucking 200 μL every 3 min the whole step took 15 min to be completed.

### Device functionalization with UV activated antibodies

The gold electrode of quartz crystal was functionalized with the above anti-*Salmonella* pAbs, UV activated by Photochemical Immobilization Technique (PIT). This is a quick reproducible technique which allows the immobilization of antibodies onto gold surfaces - in an upright orientation with their binding sites exposed to the environment^39^ – thanks to the UV activation of the triad cysteine-cysteine/tryptophan^45^ (typical structural characteristic of the antibodies)^46^. Briefly, the UV photon is absorbed by the tryptophan and this phenom generates solvated electrons, which are captured by electrophilic molecules such as cysteine, causing the cleavage of the disulphide bridge. The free thiol (SH) groups - generated by this last reaction – can easily interact with thiol reactive surfaces like gold electrodes through covalent bond. The increasing number of the free SH groups allows a new structural conformation of the immobilized immunoglobulin which are characterized by a good exposure of the antigen binding sites, thus significantly improving sensor sensitivity^39^.

In this experiment the PIT was carried out by irradiating a solution of 50 µg/mL anti-*Salmonella* pAbs for 1 min by a HERAEUS amalgam type NNI 40/20 lamp emitting at 254 nm with a power average of 40 W. The lamp was approximately 20 cm long and had a diameter of 1 cm, so the effective irradiation intensity for the antibodies activation was about 0.7 W/cm^2^. It is worth mentioning that PIT does not affect the ability of antibodies to capture the antigen, as reported by Funari *et al*.^47^.

### Detection of *Salmonella* in chicken meat samples

Chicken meat samples were purchased from a local supermarket and tested to confirm *Salmonella* absence, according to ISO 6579-1: 2017. Later, a test portion (25 g) was contaminated or not with *S*. Typhimurium at different concentrations ranging from 10^0^ CFU/mL to 10^5^ CFU/mL and were placed in sterile stomacher bag containing 225 mL of buffered peptone water (BPW; A.E.S. Laboratoire Groupe, France). Samples bag were then placed into a stomacher device for 90 sec. and incubated for 1 or 2 hours at 37 °C (pre-enrichment step). After the incubation, 15 mL of each sample were collected and centrifuged at 2000 rpm for 15 min to remove part of food debris. 1 mL of supernatant, containing bacterial cells, was centrifuged at 13400 rpm for 5 min at room temperature. The bacterial pellet was suspended in pure PBS and used for QCM analysis.

The measurements have been carried out by following the steps listed below (see Fig. 1a).

1. Stabilization with PBS 1X solution to stabilize the frequency of the device;
2. Functionalization with anti-*Salmonella* pAbs (50 µg/mL), previously UV photoactivated, to functionalize the gold surface;
3. Washing with PBS 1X to remove the antibodies no tethered on gold surface, so checking for the efficiency of the functionalization;
4. Blocking with BSA (50 µg/mL) to hold non-specific sites and avoiding free spaces onto gold surface;
5. Sample analysis of chicken meat infected or not with *S*. Typhimurium or *E. coli* or both, for testing the biosensor response;
6. Washing with PBS 1X to remove all the elements which have no interacted with the antibodies or with gold surface.

Each of the steps entailed the change of 1 mL volume and was carried out in 15 min.

At each chicken sample flowed into circuit corresponds a variation of the resonance frequency and this correlation has been explained by Sauerbrey equation^37^. This equation states that the decrease in frequency is linearly proportional to the increase in surface mass loading of QCM. Data collected were fitted to obtain a dose-response curve.

Before the pre-enrichment step, the samples contaminated or not were diluted and then spotted on Salmonella Chromogenic Agar Base (CM1007, Oxoid, Thermo Fisher, UK) or Tryptone Bile X-Gluc (TBX Agar, CM0945, Oxoid, Thermo Fisher, UK) for *S*. Typhimurium or *E. coli* isolation, respectively. The plates were then incubated at 37 °C (*S*. Typhimurium) or 44 °C (*E. coli*) for 24 h. This procedure allowed us to validate the presence and the concentration of the bacteria in food samples.

### Method specificity

In order to evaluate the method specificity, the chicken meat samples were infected with *E. coli* (10^4^ CFU/mL) alone or in combination with *S*. Typhimurium (both bacteria at 10^4^ CFU/mL). All the sample infected were pre-enriched for 2 hours at 37 °C and later, analysed at QCM immunosensor.

### Statistics

Specificity of anti-*Salmonella* pAbs was assessed using ANOVA Test (GraphPad Software, La Jolla, CA, USA) and only differences with p-value < 0.05 were considered significant.

## References

[1] Grimont, P. & Weill, F. -X. Antigenic Formulae of the Salmonella Servovars. *WHO Collab. Cent. Ref. Res. Salmonella*, 1–167 (2008).

[2] Food, E. & Authority, S. The European Union Summary Report on Trends and Sources of Zoonoses, Zoonotic Agents and Food‐borne Outbreaks in 2015. *EFSA J*. **14** (12) (2016). 10.2903/j.efsa.2017.5077 PMC7009962 32625371

[3] Havelaar AH (2012). Disease Burden of Foodborne Pathogens in the Netherlands, 2009. Int. J. Food Microbiol..

[4] Jurado-Tarifa E (2016). Genetic Diversity and Antimicrobial Resistance of Campylobacter and Salmonella Strains Isolated from Decoys and Raptors. Comp. Immunol. Microbiol. Infect. Dis..

[5] Hanning IB, Nutt JD, Ricke SC (2009). Salmonellosis Outbreaks in the United States due to Fresh Produce: Sources and Potential Intervention Measures. Foodborne Pathog. Dis..

[6] Sivapalasingam S, Friedman CR, Cohen L, Tauxe RV (2004). Fresh Produce: A Growing Cause of Outbreaks of Foodborne Illness in the United States, 1973 through 1997. J. Food Prot..

[7] Mrema N, Mpuchane S, Gashe BA (2006). Prevalence of Salmonella in Raw Minced Meat, Raw Fresh Sausages and Raw Burger Patties from Retail Outlets in Gaborone, Botswana. Food Control.

[8] Crum-Cianflone NF (2008). Salmonellosis and the Gastrointestinal Tract: More than Just Peanut Butter. Curr. Gastroenterol. Rep..

[9] Rai M, Pandit R, Gaikwad S, Kövics G (2016). Antimicrobial Peptides as Natural Bio-Preservative to Enhance the Shelf-Life ofFood. J. Food Sci. Technol..

[10] Capparelli Rosanna, De Chiara Francesco, Nocerino Nunzia, Montella Rosa Chiara, Iannaccone Marco, Fulgione Andrea, Romanelli Alessandra, Avitabile Concetta, Blaiotta Giuseppe, Capuano Federico (2012). New perspectives for natural antimicrobial peptides: application as antinflammatory drugs in a murine model. BMC Immunology.

[11] ISO. ISO 11290-1:1996 Microbiology of Food and Animal Feeding Stuffs - Horizontal Method for the Detection and Enumeration of Listeria Monocytogenes - Part 1: Detection Method. *International Organization for Standardization, Geneva*., p 16 (1996).

[12] Evers EG (2004). Predicted Quantitative Effect of Logistic Slaughter on Microbial Prevalence. Prev. Vet. Med..

[13] Delibato E (2013). Validation of a 1-Day Analytical Diagnostic Real-Time PCR for the Detection of Salmonella in Different Food Meat Categories. Food Anal. Methods.

[14] Lee Kyung-Min, Runyon Mick, Herrman Timothy J., Phillips Robert, Hsieh John (2015). Review of Salmonella detection and identification methods: Aspects of rapid emergency response and food safety. Food Control.

[15] Eijkelkamp JM, Aarts HJM, Van Der Fels-Klerx HJ (2009). Suitability of Rapid Detection Methods for Salmonella in Poultry Slaughterhouses. Food Anal. Methods.

[16] López-Campos, G., Martínez-Suárez, J. V., Aguado-Urda, M. & López-Alonso, V. Microarray Detection and Characterization of Bacterial Foodborne Pathogens. *Food, Heal. Nutr*. 13–33 (2012).

[17] Mortari Alessia, Lorenzelli Leandro (2014). Recent sensing technologies for pathogen detection in milk: A review. Biosensors and Bioelectronics.

[18] Leonard Paul, Hearty Stephen, Brennan Joanne, Dunne Lynsey, Quinn John, Chakraborty Trinad, O’Kennedy Richard (2003). Advances in biosensors for detection of pathogens in food and water. Enzyme and Microbial Technology.

[19] Park M-K, Oh J-H (2012). Rapid Detection of *E. Coli* O157:H7 on Turnip Greens Using a Modified Gold Biosensor Combined with Light Microscopic Imaging System. J. Food Sci..

[20] Ricci Francesco, Volpe Giulia, Micheli Laura, Palleschi Giuseppe (2007). A review on novel developments and applications of immunosensors in food analysis. Analytica Chimica Acta.

[21] Shu Jian, Tang Dianping (2017). Current Advances in Quantum-Dots-Based Photoelectrochemical Immunoassays. Chemistry - An Asian Journal.

[22] Zhang K, Lv S, Lin Z, Tang D (2017). CdS:Mn Quantum Dot-Functionalized G-C3N4nanohybrids as Signal-Generation Tags for Photoelectrochemical Immunoassay of Prostate Specific Antigen Coupling DNAzyme Concatamer with Enzymatic Biocatalytic Precipitation. Biosens. Bioelectron..

[23] Zhang K, Lv S, Lin Z, Li M, Tang D (2018). Bio-Bar-Code-Based Photoelectrochemical Immunoassay for Sensitive Detection of Prostate-Specific Antigen Using Rolling Circle Amplification and Enzymatic Biocatalytic Precipitation. Biosens. Bioelectron..

[24] Lin Y, Zhou Q, Tang D, Niessner R, Knopp D (2017). Signal-On Photoelectrochemical Immunoassay for Aflatoxin B 1 Based on Enzymatic Product-Etching MnO_2_ Nanosheets for Dissociation of Carbon Dots. Anal. Chem..

[25] Lai W, Wei Q, Xu M, Zhuang J, Tang D (2017). Enzyme-Controlled Dissolution of MnO2nanoflakes with Enzyme Cascade Amplification for Colorimetric Immunoassay. Biosens. Bioelectron..

[26] Mazumdar SD, Barlen B, Kämpfer P, Keusgen M (2010). Surface Plasmon Resonance (SPR) as a Rapid Tool for Serotyping of Salmonella. Biosens. Bioelectron..

[27] Xiang C (2015). Sensitive Electrochemical Detection of Salmonella with Chitosan-Gold Nanoparticles Composite Film. Talanta.

[28] Fei J, Dou W, Zhao G (2015). A Sandwich Electrochemical Immunosensor for Salmonella Pullorum and Salmonella Gallinarum Based on a Screen-Printed Carbon Electrode Modified with an Ionic Liquid and Electrodeposited Gold Nanoparticles. Microchim. Acta.

[29] Funari Riccardo, Della Ventura Bartolomeo, Carrieri Raffaele, Morra Luigi, Lahoz Ernesto, Gesuele Felice, Altucci Carlo, Velotta Raffaele (2015). Detection of parathion and patulin by quartz-crystal microbalance functionalized by the photonics immobilization technique. Biosensors and Bioelectronics.

[30] Della Ventura B (2017). Effective Antibodies Immobilization and Functionalized Nanoparticles in a Quartz-Crystal Microbalance-Based Immunosensor for the Detection of Parathion. PLoS One.

[31] Waiwijit, U. *et al*. Biosensor for Salmonella Typhimurium Detection. In *2012 IEEE International Conference on Electron Devices and Solid State Circuit, EDSSC 2012* (2012).

[32] Su XL, Li YA (2005). QCM Immunosensor for Salmonella Detection with Simultaneous Measurements of Resonant Frequency and Motional Resistance. Biosens. Bioelectron..

[33] Kim G, Moon JH, Moh CY, Lim J (2015). guk. A Microfluidic Nano-Biosensor for the Detection of Pathogenic Salmonella. Biosens. Bioelectron..

[34] Salam F, Uludag Y, Tothill IE (2013). Real-Time and Sensitive Detection of Salmonella Typhimurium Using an Automated Quartz Crystal Microbalance (QCM) Instrument with Nanoparticles Amplification. Talanta.

[35] Wang L (2017). QCM-Based Aptamer Selection and Detection of Salmonella Typhimurium. Food Chem..

[36] Kanazawa KK, Gordon JG (1985). Frequency of a Quartz Microbalance in Contact with Liquid. Anal. Chem..

[37] Sauerbrey GV (1959). von Schwingquarzen Zur Wägung Dünner Schichten Und Zur Mikrowägung. Zeitschrift für Phys..

[38] Rolfe MD (2012). Lag Phase Is a Distinct Growth Phase That Prepares Bacteria for Exponential Growth and Involves Transient Metal Accumulation. J. Bacteriol..

[39] Funari R (2016). Single Molecule Characterization of UV-Activated Antibodies on Gold by Atomic Force Microscopy. Langmuir.

[40] de Boer E, Beumer RR (1999). Methodology for Detection and Typing of Foodborne Microorganisms. Int. J. Food Microbiol..

[41] Ivnitski D, Abdel-Hamid I, Atanasov P, Wilkins E, Stricker S (2000). Application of Electrochemical Biosensors for Detection of Food Pathogenic Bacteria. Electroanalysis.

[42] Müller-Loennies, S., Brade, L. & Brade, H. Neutralizing and Cross-Reactive Antibodies against Enterobacterial Lipopolysaccharide. I*nternational Journal of Medical Microbiology*. pp 321–340 (2007). 10.1016/j.ijmm.2007.04.002 17544324

[43] Singh SP, Upshaw Y, Abdullah T, Singh SR, Klebba PE (1992). Structural Relatedness of Enteric Bacterial Porins Assessed with Monoclonal Antibodies to Salmonella Typhimurium OmpD and OmpC. J. Bacteriol..

[44] Keasey SL (2009). Extensive Antibody Cross-Reactivity among Infectious Gram-Negative Bacteria Revealed by Proteome Microarray Analysis. Mol. Cell. Proteomics.

[45] Neves-Petersen MT (2002). High Probability of Disrupting a Disulphide Bridge Mediated by an Endogenous Excited Tryptophan Residue. Protein Sci..

[46] Ioerger TR, Du C, Linthicum DS (1999). Conservation of Cys-Cys Trp Structural Triads and Their Geometry in the Protein Domains of Immunoglobulin Superfamily Members. Mol. Immunol..

[47] Funari R (2013). Detection of Parathion Pesticide by Quartz Crystal Microbalance Functionalized with UV-Activated Antibodies. Anal. Chem..

